# The Temporal Lobes Differentiate between the Voices of Famous and Unknown People: An Event-Related fMRI Study on Speaker Recognition

**DOI:** 10.1371/journal.pone.0047626

**Published:** 2012-10-24

**Authors:** Anja Bethmann, Henning Scheich, André Brechmann

**Affiliations:** 1 Special Lab Non-Invasive Brain Imaging, Leibniz Institute for Neurobiology, Magdeburg, Germany; 2 Auditory Learning & Speech, Leibniz Institute for Neurobiology, Magdeburg, Germany; 3 Special Lab Non-Invasive Brain Imaging, Leibniz Institute for Neurobiology, Magdeburg, Germany; University Of Cambridge, United Kingdom

## Abstract

It is widely accepted that the perception of human voices is supported by neural structures located along the superior temporal sulci. However, there is an ongoing discussion to what extent the activations found in fMRI studies are evoked by the vocal features themselves or are the result of phonetic processing. To show that the temporal lobes are indeed engaged in voice processing, short utterances spoken by famous and unknown people were presented to healthy young participants whose task it was to identify the familiar speakers. In two event-related fMRI experiments, the temporal lobes were found to differentiate between familiar and unfamiliar voices such that named voices elicited higher BOLD signal intensities than unfamiliar voices. Yet, the temporal cortices did not only discriminate between familiar and unfamiliar voices. Experiment 2, which required overtly spoken responses and allowed to distinguish between four familiarity grades, revealed that there was a fine-grained differentiation between all of these familiarity levels with higher familiarity being associated with larger BOLD signal amplitudes. Finally, we observed a gradual response change such that the BOLD signal differences between unfamiliar and highly familiar voices increased with the distance of an area from the transverse temporal gyri, especially towards the anterior temporal cortex and the middle temporal gyri. Therefore, the results suggest that (the anterior and non-superior portions of) the temporal lobes participate in voice-specific processing independent from phonetic components also involved in spoken speech material.

## Introduction

Some years ago, it was suggested that areas in the temporal lobes are involved in the processing of other person’s voices. It was found that the perception of such voices evoked widespread neural activation bilaterally in the superior and middle temporal cortex of the human brain when these stimuli were contrasted with rest periods [1], with the perception of faces [2], [3], or with meaningless acoustic control stimuli [2], [4]–[6]. Most of these studies noticed activation centres within the transverse temporal gyri or in adjacent areas, but more anterior portions of the superior temporal gyri (STG) and areas around the superior temporal sulci (STS) were also frequently reported to host activation peaks. That the anterior STG and areas along the STS are of significance for voice processing is supported by studies that compared the perception of human voices and meaningful environmental sounds. In those studies, activations peaks were predominantly located in the upper bank of the STS all along its horizontal length [4], [7], [8]. As these areas preferentially responded to human voices, they were termed ‘voice-selective areas’ [7].

Other studies challenged the view that these areas surrounding the STS serve a ‘voice-selective’ function. It was found that these parts of the temporal lobes were strongly activated even if the subjects’ task was to focus on linguistic aspects of spoken speech material, either in comparison to meaningless sounds [9]–[12] or compared with meaningful environmental sounds [13]–[17]. These results were interpreted as evidence for a specific role of these brain regions in linguistic processing. Yet, some data cannot be explained by linguistic processing alone. When pure voice processing was probed, activation centres were still identified in areas around the STS. This was achieved by presenting non-speech vocalisations to inhibit linguistic processing such as laughs, sighs, or coughs [4], [5] or by directly contrasting voice and speech processing [1], [6], [18]–[23]. With this kind of studies, the activation centres were again distributed all over the STS. But, in contrast to the before-mentioned studies, there was a preponderance of clusters that was located in the anterior temporal cortex (ATL) or, to be more precise, in the anterior part of the STG near the STS.

If the assumption is correct that areas along the STS are relevant to voice processing, one might ask whether these regions help to discriminate between familiar and unfamiliar voices. To date, there are only a few studies that directly compared the neural processing of familiar and unfamiliar voices. One such study reported that higher signal intensities to familiar voices were restricted to an area outside of the temporal lobes (to the retrosplenial cortex) [3]. Another study observed activation differences within the temporal lobes, namely in the central-posterior STG/STS, but with stronger activation by unfamiliar voices [22]. With a learning paradigm, Latinus et al. [24] found a stronger signal before than after voice learning in the right anterior STG. Yet other studies identified higher signals in the temporal lobes for familiar than unfamiliar voices, one in several parts of both temporal lobes including the right temporal pole, anterior inferior temporal regions, anterior medial temporal areas, temporo-occipito-parietal cortices, and the fusiform cortex [23], the other in the central-anterior middle temporal gyrus (MTG) of the left hemisphere only [25]. Finally, Nakamura et al. [19] compared a familiarity decision task on familiar and unfamiliar voices to a phonetic processing task using voices of unfamiliar people only. The authors found that the familiarity task caused stronger neural activation in the right temporal pole and noticed that the signal in that area significantly correlated to the number of the identified speakers. Thus, the studies did not clarify unequivocally whether or not the temporal lobes differentiate between familiar and unfamiliar voices.

Therefore, the present study was designed to examine the neural response in the temporal lobes when healthy young participants identify famous and unknown speakers by their voices. The activation in response to voices of different familiarity levels was analysed by means of a region-of-interest approach that subdivided each temporal lobe into several subregions. That way, the study aimed at contributing to three questions: Does the intensity of the BOLD signal in the temporal lobes differ between familiar and unfamiliar voices? Is the BOLD intensity higher with familiar or with unfamiliar voices? And which temporal regions distinguish most clearly between familiar and unfamiliar voices?

## Results

### Behavioural Data

The speaker recognition tasks of both experiments resulted in a very low number of recognised or correctly named voices. In Experiment 1, most of the 75 voices, viz. 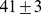, were rated as being unfamiliar (U). Further 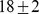 were rated as being familiar, but could not be identified unequivocally (F). Successful name retrieval was indicated for 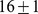 out of the 50 voices being from famous individuals (N). Thus, the number of unfamiliar, familiar, and named voices differed significantly (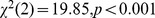, Friedman). Pairwise comparisons revealed that significantly more voices were classified as being unfamiliar than as being familiar or named (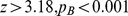, Wilcoxon). The number of familiar and named voices, however, was broadly similar (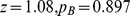).

Also in Experiment 2, most of the 80 voices presented, viz. 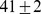, were classified as being unfamiliar (U). Another 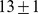 voices sounded familiar to the subjects who could not provide further details about the speakers (F). 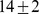 stimuli were associated with a particular person but could not be named (A). Association was assumed when subjects gave a semantic description or produced a false name. Only 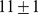 out of 70 famous voices were named correctly (N). Again, the number of unfamiliar, familiar, associated, and named voices differed significantly (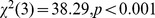, Friedman). Yet, this only holds for the unfamiliar compared with the more familiar voices. Pairwise comparisons revealed that there were no significant differences between the number of familiar, associated, and named voices (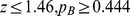, Wilcoxon). These response types, however, were given less often than classifying a voice as being unfamiliar (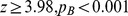). Hence, the recognition rate was low in both experiments with 32% in the first experiment and 16% in the second one. Yet, it was comparable to other studies, which found that the voice recognition performance is generally in the range of 15–35% [26]–[30].

**Figure 1:**
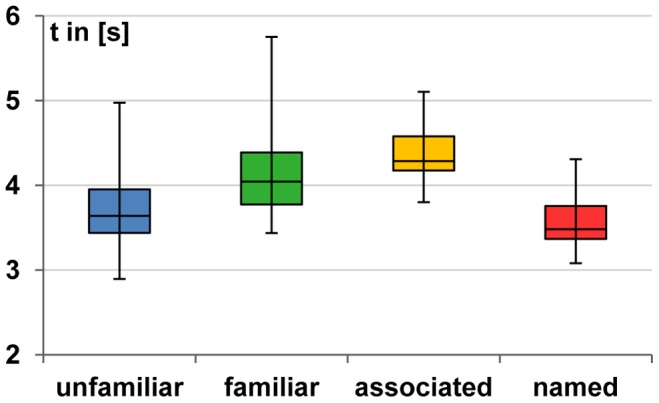
Experiment 2– Response times to indicate voice familiarity by overt speaking. Each box gives the median with the 1st and 3rd quartile, the whiskers show the minimum and maximum values.

In contrast to the first experiment, the paradigm of Experiment 2 allowed to analyse and compare the different response times (see Tasks for details). Response time was the time span from stimulus onset to the onset of a spoken response. The response time was found to vary as a function of voice type (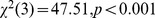, Friedman; see Figure 1). Pairwise comparisons revealed that the response times to unfamiliar (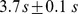) and named (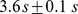) voices were significantly shorter than to familiar (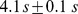) and associated (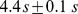) voices (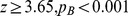, Wilcoxon). The latter two types also differed in their reaction times (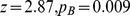). The response times to unfamiliar and named voices, however, did not differ significantly (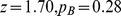).

### Imaging Results

The processing of human voices evoked a widespread neural activation within the temporal lobes of all participants. In Experiment 1, the main condition *Named Voices* (compared to rest) resulted in a mean of 12 724 activated voxels across all left-sided regions-of-interest (ROIs) and of 14 680 voxels across all right-sided ROIs at the statistical level of 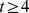. Each voxel had a size of 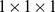 mm^3^. The respective volumes for Experiment 2 were 16 970 mm^3^ and 18 799 mm^3^. The named voices elicited a strong BOLD signal with intensities that were very similar in both hemispheres. In Experiment 1, the BOLD signal was about 1.1%; in Experiment 2, it was almost twice as high with 1.9%. The values of the individual ROIs are given in Table 1 for the first experiment and in Table 2 for the second experiment.

**Figure 2:**
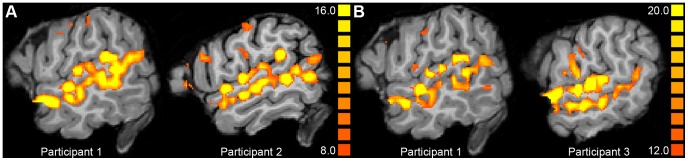
Examples of activation maps. Shown for the contrast Voices > Rest at the significance level of 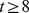 (Experiment 1, A) or 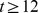 (Experiment 2, B) in different participants shown in sagittal plane at 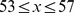.

Using a stricter statistical threshold than was applied for analysis, it was found that the activation of the temporal lobes in response to human voices was composed of numerous activation clusters, as can be seen in Figure 2. Activated areas involved the upper and lower banks of the STS where the activation clusters neatly lined up from one end to the other.

**Table 1:** Experiment 1– (a) Number of activated voxels for the contrast named voices 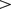 rest, (b–d) BOLD intensities evoked by the different voice categories, (e) BOLD ratio of named to non-named voices.

		a		Ma		mp		p	
		LH	RH	LH	RH	LH	RH	LH	RH
(a) Voxels	**sSTG**	457	400	1810	1600	2792	2566	939	742
		130	73	193	155	261	302	162	191
	**iSTG**	273	452	1601	1806	2425	2586	950	1293
		66	92	212	166	140	243	172	303
	**sMTG**	65	165	293	692	655	1557	463	819
		18	41	73	132	121	207	97	190
(b) U	**sSTG**	0.656	0.619	1.045	1.072	1.240	1.170	0.725	0.654
		0.114	0.075	0.074	0.101	0.065	0.064	0.098	0.083
	**iSTG**	0.492	0.573	0.690	0.791	0.861	0.905	0.636	0.692
		0.045	0.069	0.039	0.051	0.051	0.067	0.061	0.089
	**sMTG**	0.281	0.429	0.377	0.511	0.513	0.778	0.455	0.735
		0.076	0.069	0.034	0.056	0.064	0.055	0.052	0.084
(c) F	**sSTG**	0.819	0.782	1.088	1.078	1.234	1.142	0.757	0.637
		0.093	0.058	0.065	0.087	0.073	0.069	0.107	0.079
	**iSTG**	0.609	0.631	0.677	0.813	0.861	0.928	0.642	0.711
		0.079	0.063	0.037	0.046	0.050	0.062	0.053	0.085
	**sMTG**	0.433	0.460	0.384	0.531	0.544	0.791	0.407	0.730
		0.099	0.057	0.051	0.071	0.069	0.069	0.037	0.073
(d) N	**sSTG**	1.117	0.995	1.215	1.229	1.351	1.260	0.937	0.786
		0.072	0.074	0.076	0.097	0.074	0.070	0.098	0.072
	**iSTG**	0.898	0.813	0.915	0.984	1.054	1.045	0.877	0.888
		0.099	0.070	0.046	0.061	0.049	0.050	0.062	0.080
	**sMTG**	0.775	0.789	0.696	0.747	0.827	1.003	0.712	0.924
		0.137	0.099	0.043	0.059	0.066	0.063	0.050	0.076
(e) N > UF	**sSTG**	34	28	12	13	8	8	23	19
		6	5	2	2	1	2	4	4
	**iSTG**	35	25	24	18	18	13	27	23
		7	4	3	3	3	2	2	4
	**sMTG**	50	41	43	30	36	22	39	21
		12	7	6	5	6	4	5	4

Given are the mean and in small numbers the standard error of the mean. Abbreviations: *a*, anterior; *F*, familiar; *iSTG*, inferior part of the STG; *LH*, left hemisphere; *ma*, mid-anterior; *mp*, mid-posterior; *N*, named; *p*, posterior; *RH*, right hemisphere; *sMTG*, superior part of the MTG; *sSTG*, superior part of the STG; *U*, unfamiliar.

#### Differences between conditions

The main finding from the experiments was that the intensity of the BOLD signal in the temporal lobes was strongly dependent on the familiarity of a presented voice. More precisely, familiar voices elicited higher signal intensities than less familiar voices. In Table 1 (b–c) and Table 2 (b–g) the extracted BOLD values of each condition are given for the first and second experiment, respectively.

**Table 2:** Experiment 2– (a) Number of activated voxels for the contrast named voices > rest, (b–g) BOLD intensities evoked by the different voice categories, (h) BOLD ratio of named to non-named voices.

		a		ma		mp		p	
		LH	RH	LH	RH	LH	RH	LH	RH
(a) Voxels	**sSTG**	677	660	2156	2076	3252	3250	1497	1202
		111	122	166	157	186	185	206	178
	**iSTG**	535	589	1780	2153	2733	3089	1476	1680
		81	103	189	186	213	202	209	216
	**sMTG**	247	291	614	956	968	1727	1036	1127
		56	56	139	147	155	221	169	160
(b) U	**sSTG**	1.054	1.252	1.668	1.965	2.026	2.109	1.272	1.062
		0.068	0.119	0.097	0.171	0.114	0.143	0.129	0.135
	**iSTG**	0.769	1.113	1.148	1.404	1.449	1.478	1.064	1.337
		0.067	0.090	0.085	0.079	0.101	0.070	0.116	0.146
	**sMTG**	0.482	0.628	0.649	0.923	0.908	1.358	0.732	0.927
		0.063	0.074	0.106	0.089	0.101	0.103	0.066	0.066
(c) F	**sSTG**	1.324	1.452	1.817	2.172	2.219	2.322	1.438	1.232
		0.094	0.151	0.108	0.183	0.135	0.157	0.129	0.154
	**iSTG**	0.943	1.407	1.202	1.511	1.573	1.628	1.170	1.406
		0.068	0.106	0.094	0.082	0.117	0.073	0.119	0.142
	**sMTG**	0.646	0.821	0.691	1.035	1.103	1.485	0.862	0.989
		0.098	0.089	0.113	0.128	0.143	0.111	0.103	0.087
(d) A	**sSTG**	1.680	1.837	1.972	2.301	2.363	2.459	1.533	1.349
		0.128	0.145	0.108	0.178	0.121	0.147	0.120	0.145
	**iSTG**	1.139	1.548	1.351	1.661	1.703	1.720	1.304	1.594
		0.100	0.099	0.079	0.087	0.100	0.073	0.102	0.141
	**sMTG**	0.857	0.958	0.923	1.196	1.272	1.630	1.058	1.204
		0.097	0.068	0.086	0.102	0.120	0.106	0.081	0.063
(e) N	**sSTG**	2.097	2.082	2.176	2.410	2.466	2.509	1.739	1.443
		0.127	0.135	0.136	0.199	0.147	0.169	0.165	0.145
	**iSTG**	1.310	1.710	1.601	1.823	1.864	1.850	1.569	1.763
		0.087	0.138	0.138	0.134	0.133	0.116	0.170	0.170
	**sMTG**	0.995	1.227	1.236	1.481	1.501	1.768	1.316	1.414
		0.091	0.110	0.187	0.199	0.167	0.119	0.137	0.077
(f) N_S_	**sSTG**	2.223	2.112	2.249	2.496	2.546	2.596	1.835	1.564
		0.169	0.121	0.149	0.229	0.149	0.186	0.191	0.187
	**iSTG**	1.364	1.745	1.687	1.908	1.929	1.897	1.655	1.834
		0.100	0.123	0.173	0.161	0.142	0.127	0.176	0.197
	**sMTG**	1.006	1.284	1.329	1.525	1.642	1.796	1.421	1.447
		0.089	0.119	0.259	0.210	0.212	0.126	0.157	0.091
(g) N_Q_	**sSTG**	1.961	2.027	2.112	2.342	2.388	2.437	1.667	1.355
		0.116	0.159	0.136	0.188	0.150	0.163	0.158	0.131
	**iSTG**	1.250	1.672	1.536	1.755	1.806	1.809	1.488	1.711
		0.087	0.162	0.121	0.122	0.129	0.113	0.172	0.160
	**sMTG**	0.980	1.173	1.182	1.446	1.388	1.733	1.233	1.377
		0.109	0.108	0.156	0.200	0.144	0.117	0.132	0.074
(h) N > UF	**sSTG**	40	33	19	14	13	11	22	24
		4	5	2	2	2	2	3	3
	**iSTG**	34	25	26	18	19	15	29	23
		4	3	2	2	2	2	3	2
	**sMTG**	46	40	48	31	33	20	37	33
		4	4	4	4	4	2	5	4

Given are the mean and in small numbers the standard error of the mean. Abbreviations: *A*, associated; *a*, anterior; *F*, familiar; *iSTG*, inferior part of the STG; *ma*, mid-anterior; *mp*, mid-posterior; *N*, named; *N_Q_*, named quickly, *N_S_*, named slowly; *p*, posterior; *sMTG*, superior part of the MTG; *sSTG*, superior part of the STG; *U*, unfamiliar.

**Figure 3:**
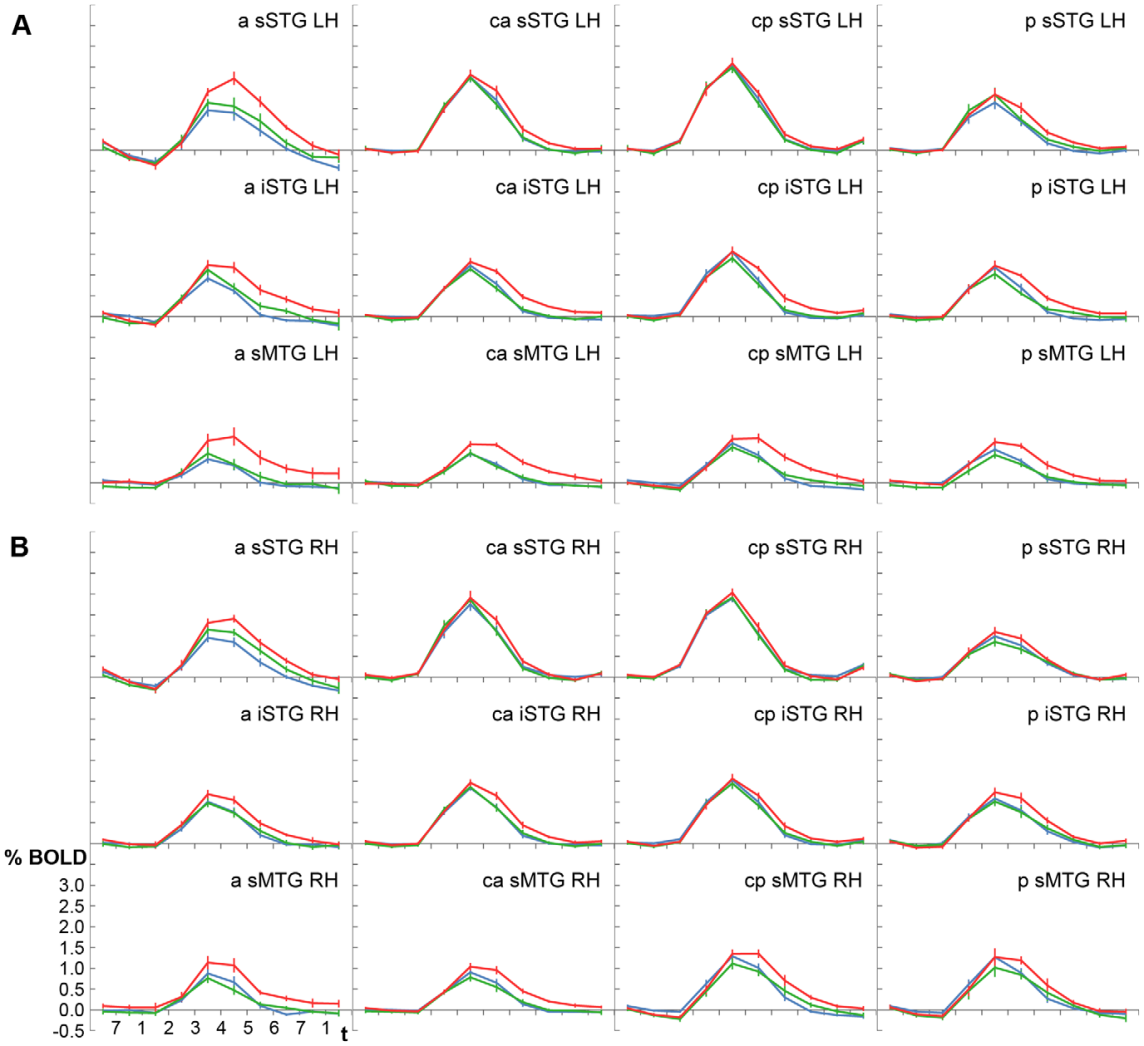
Experiment 1– Averaged BOLD signal time courses shown for all regions-of-interest in the left (A) and right (B) hemisphere. Time t is given as the duration to scan one volume (2 s). Blue =  unfamiliar voices, green =  familiar voices, red =  named voices. Regions: a, anterior; ma, mid-anterior; mp, mid-posterior; iSTG, inferior part of the STG; LH, left hemisphere; p, posterior; RH, right hemisphere; sMTG, superior part of the MTG; sSTG, superior part of the STG.

In Experiment 1, the BOLD signal evoked by the unfamiliar (U), familiar (F), and named (N) voices was significantly different in all temporal ROIs (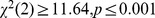, Friedman). Post hoc tests confirmed that named voices resulted in a stronger signal than unfamiliar and familiar voices (Figure 3). The pairwise comparisons identified significant signal differences for N > U and for N > F in 23 of the 24 ROIs (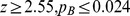, Wilcoxon). Both contrasts did not reach significance in one ROI each. This was ROI *mp-sSTG-RH* for the contrast N > U (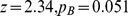) and ROI *a-sMTG-LH* for N > F (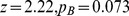). Across all temporal ROIs, the differences between named and non-named voices had a magnitude of about 0.15–0.20%. In contrast, there were only minor signal differences between unfamiliar and familiar voices of approximately 0.01%. These two conditions did not differ significantly from one another in any ROI (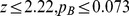).

**Figure 4:**
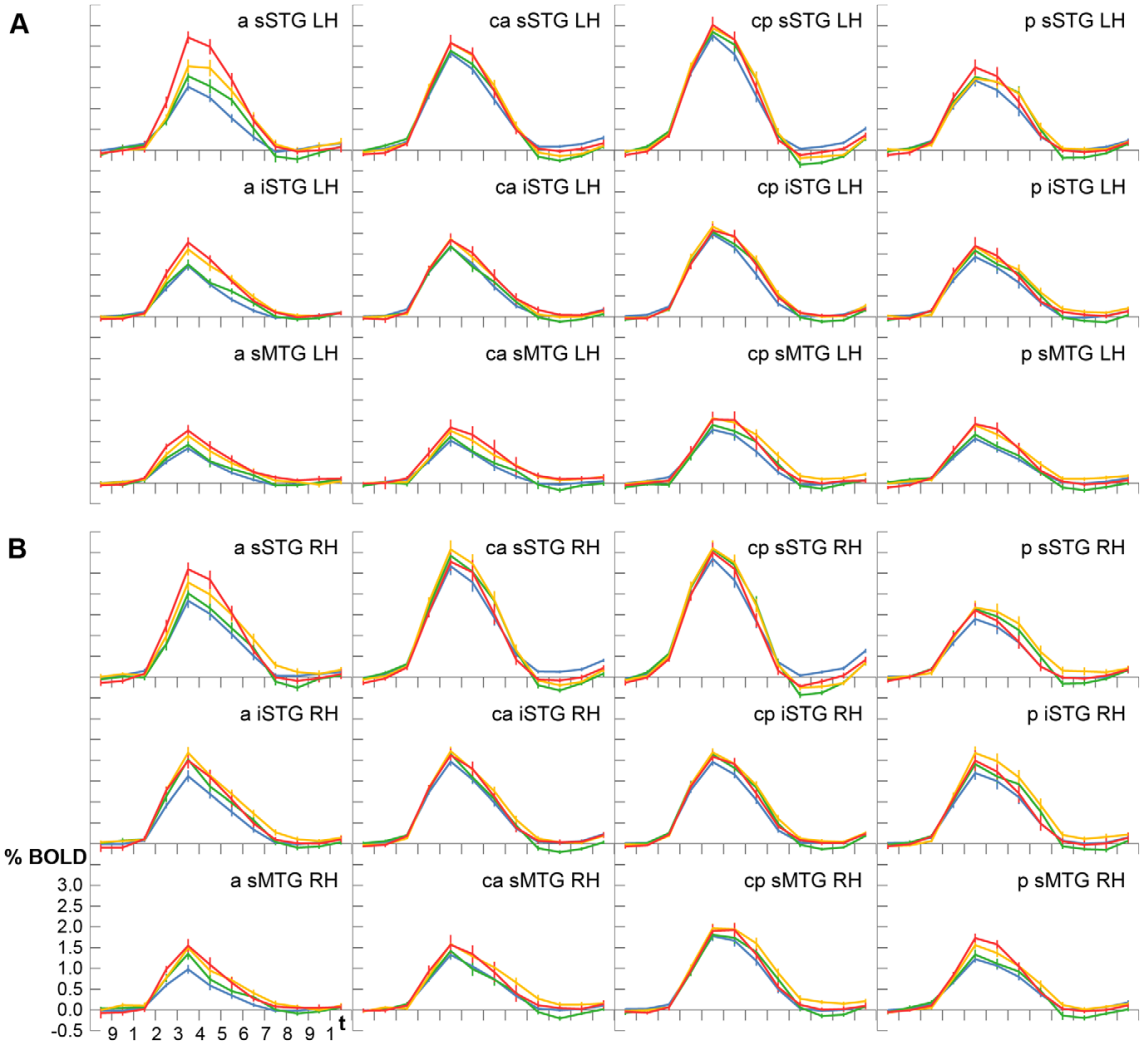
Experiment 2– Averaged BOLD signal time courses shown for all regions-of-interest in the left (A) and right (B) hemisphere. Blue =  unfamiliar voices, green =  familiar voices, yellow =  associated voices, red =  named voices. For abbreviations and further information see Figure 3.

Experiment 2 confirmed that the intensity of the BOLD signal varies depending on voice familiarity. Significant differences were again observed in each ROI (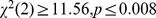, Friedman). Yet, due to the overt response paradigm, which allowed to distinguish four different familiarity states, the gradation could be observed to be more fine-grained than in the first experiment. Pairwise comparisons showed that unfamiliar voices (U) resulted in a weaker signal than voices that caused some familiarity feelings (F), both generated weaker neural activity than associated voices (A), and all three types a weaker signal than correctly named voices (N; Figure 4). This order of signal intensities U < F < A < N was evident in many ROIs and reversed in none (Table 3). The signal difference between similar familiarity grades as F > U, A > F, or N > A was in the magnitude of 0.12–0.20%, between more different familiarity levels as A > U or N > F of 0.27–0.35%, and for N > U of more than 0.42% across all ROIs. Additionally, the signal strength was observed to vary according to the reaction times. In ROIs with a significant signal difference, slowly named voices evoked higher signal intensities than quickly named voices. The difference across all ROIs had a magnitude of approximately 0.13%.

**Table 3:** Experiment 2– Differences in the BOLD signal intensities between conditions.

	a		ma		mp		p	
	LH	RH	LH	RH	LH	RH	LH	RH
F > U								
**sSTG**	+		+	+	+	+		+
**iSTG**	+	+				+		
**sMTG**		+			+			
A > U								
**sSTG**	+	+	+	+	+	+	+	+
**iSTG**	+	+	+	+	+	+	+	+
**sMTG**	+	+	+	+	+	+	+	+
A > F								
**sSTG**	+	+		+		+		
**iSTG**	+		+	+			+	+
**sMTG**	+		+	+	+	+	+	+
N > U								
**sSTG**	+	+	+	+	+	+	+	+
**iSTG**	+	+	+	+	+	+	+	+
**sMTG**	+	+	+	+	+	+	+	+
N > F								
**sSTG**	+	+	+	+	+		+	+
**iSTG**	+	+	+	+	+	+	+	+
**sMTG**	+	+	+	+	+	+	+	+
N > A								
**sSTG**	+		+				+	
**iSTG**	+		+		+	+	+	+
**sMTG**		+	+	+			+	+
N_S_ > N_Q_								
**sSTG**	+		+	+	+	+	+	+
**iSTG**			+	+	+		+	+
**sMTG**					+		+	

Regions with significant BOLD differences between two voice categories were marked by a plus sign (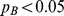, Wilcoxon, Bonferroni adjusted). Abbreviations: *A*, associated; *a*, anterior; *F*, familiar; *iSTG*, inferior part of the STG; *ma*, mid-anterior; *mp*, mid-posterior; *N*, named; *N_Q_*, named quickly, *N_S_*, named slowly; *p*, posterior; *sMTG*, superior part of the MTG; *sSTG*, superior part of the STG; *U*, unfamiliar.

#### Differences between ROIs

As described in the previous section, voices of higher familiarity evoked larger BOLD signal intensities in the temporal lobes than less familiar or unfamiliar voices. The largest differences between disparate voice categories were observed between named and unfamiliar voices. Yet, the differences were not of the same magnitude in all regions-of-interest. In both experiments, they were found to be small in central parts of the STG and larger in ROIs that were located in more anterior, more posterior, or more inferior temporal regions (see Figure 3 and Figure 4). In contrast, the signal differences between less familiar and unfamiliar voices (F > U or A > U) remained fairly stable across ROIs.

The signal differences between very and faintly familiar voices were captured by calculating the BOLD ratio of named to unfamiliar and familiar-only voices (N > UF) using Equation 1. The BOLD ratio for each ROI is given in row (e) of Table 1 for the first experiment and in row (h) of Table 2 for the second experiment. Low values indicate that there were only minor signal differences between named and non-named voices, high values signify that the differences were especially large. The Friedman test confirmed significant differences in the BOLD ratio across ROIs along the anterior-posterior axis and along the superior-inferior axis. In Experiment 2, all 14 comparisons reached significance (each hemisphere; anterior-posterior axis: sSTG, iSTG, and sMTG with 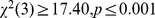; superior-inferior axis: anterior, mid-anterior, mid-posterior, and posterior with 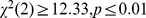). In Experiment 1, ten comparisons reached significance (anterior-posterior axis with 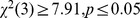; superior-inferior axis with 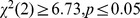; insignificant: *sMTG-LH* with 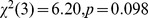, *a-RH* with 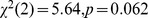, *mp-RH* with 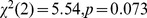, and *p-RH* with 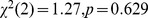). In both experiments, the lowest values for the BOLD ratio N > UF were found bilaterally in the ROIs *mp-sSTG*. From there, the signal differences between the named and non-named voices increased gradually towards more anterior, posterior, and inferior regions. Along the superior-inferior-axis, the ratio was generally lower in *sSTG*-ROIs than in *iSTG*-ROIs and they were also smaller in *iSTG*-ROIs than in *sMTG*-ROIs. Along the anterior-posterior-axis, the indices were smallest in *mp*-ROIs, followed by *ma*-ROIs, whose values were also smaller than in *p*-ROIs. The *a*-ROIs got the largest indices. The results of the pairwise comparisons are given in Table 4. To sum up, it was areas in the superior part of the MTG and in anterior portions of the temporal lobes that most clearly distinguished between named voices on the one hand and unfamiliar or familiar-only voices on the other hand.

**Table 4:** Changes in the BOLD ratio N > FU across the temporal lobes, i.e. in the signal difference between named and non-named voices.

		Left hemisphere							Right hemisphere						
		a		ma		mp		p	a		ma		mp		p
Exp. 1	**sSTG**		←				→			←		←		→	
				↓		↓									
	**iSTG**						→			→					
				↓		↓		↓	↓		↓		↓		
	**sMTG**														
Exp. 2	**sSTG**		←		←		→			←				→	
				↓		↓		↓	↓						
	**iSTG**		←		←		→			←		←		→	
		↓		↓		↓		↓	↓		↓		↓		↓
	**sMTG**				←							←		→	

Arrows indicate a significant increase in the BOLD ratio N > FU from one ROI to another (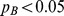, Wilcoxon, Bonferroni adjusted). Abbreviations: *a*, anterior; *iSTG*, inferior part of the STG; *ma*, mid-anterior; *mp*, mid-posterior; *p*, posterior; *sMTG*, superior part of the MTG; *sSTG*, superior part of the STG.

## Discussion

### The Temporal Lobes Respond more to Familiar than to Unfamiliar Voices

Two slow event-related fMRI experiments were performed with the aim to analyse the neural activity in the temporal lobes in response to familiar human voices. For that purpose, we presented the voices of famous people, interspersed with those of unknown people, and asked healthy young adults to perform a familiarity decision task. The main finding from the current study is that the BOLD signal in the temporal lobes differed as a function of voice familiarity, with more familiar voices evoking larger signal intensities than less familiar voices. In particular, the first experiment showed that named voices evoked a larger signal than unfamiliar and familiar-only voices. In the second experiment, overtly named voices elicited the highest and unfamiliar voices the lowest BOLD response. In between, voices that could not be named (correctly) but were associated with a particular speaker resulted in a larger BOLD signal than familiar-only voices. Therefore, the second major finding of the current study is that not only familiar and unfamiliar voices were distinguished by the temporal cortices but that these provide a fine-grained differentiation between voices of several familiarity levels.

BOLD differences between familiar and unfamiliar voices were observed both with manual (Experiment 1) and with spoken (Experiment 2) responses, which suggests that the response type had little effect on the neural activity in the temporal lobes. Also the response time had only a minor influence on the strength of the BOLD signal when different voice categories were compared to each other. In Experiment 2, for example, the response times to unfamiliar and named voices did not differ significantly from one another, but these two voice categories evoked the most pronounced signal differences. Hence, it was predominantly the familiarity with a voice/person that triggered BOLD differences, with more familiar voices eliciting increasingly higher signal intensities (see previous paragraph). This suggests that it was the amount of semantic information, which could be retrieved for a person, that determined the intensity of the BOLD signal. However, the signal intensity depended on the response time when slowly and quickly named voices were contrasted. A higher signal to the slowly named voices was observed. Continuing our argumentation, we suggest that the prolonged search for semantic or lexical information about a person enhanced the neural activity in the temporal lobes, which obviously are not only concerned with acoustical analyses but also with the retrieval of semantic information about familiar persons [31]–[33].

Although those experiments should help to specify the precise role of the ‘voice areas’ along the STS [7], [8], [18], there are to date only few studies that looked for differences in the neural processing of familiar and unfamiliar voices. And the few existent studies even obtained opposite results (see Introduction). Some reports described a higher signal in the temporal lobes in response to familiar voices ([3], [19], [23], [25], current study) and some to unfamiliar ones [22], [24]. We noticed that task demands might have caused these inconsistencies. Familiar voices evoked a significantly stronger signal only if the subjects were asked or were free to focus on the familiarity of the presented voices. In contrast, if a detailed acoustic comparison between voice samples was required, familiar voices elicited lower signals than unfamiliar voices. Latinus et al. [24] interpreted the latter finding as evidence for an acoustic-based processing of unfamiliar voices in the temporal lobes. Yet, as the data were gathered in the context of a voice learning paradigm, the results are also compatible with the explanation that training resulted in stored representations, which facilitated the same-different discriminations the subjects had to perform, thereby reducing the signal to the now familiar voices. A preference for unfamiliar voices in the context of an acoustic analysis between voice samples was also noticed as main result by von Kriegstein & Giraud [22], who presented voices of familiar and unknown people and asked the subjects to accomplish either a target voice or a target sentence recognition task. However, in a subsequent report of the very same data, von Kriegstein et al. [23] highlighted activation clusters within the temporal cortex that preferred familiar over unfamiliar voices. The authors themselves did not discuss their contradictory results, but the BOLD time curves presented in the former study showed that both patterns were already present in the first analysis. Whether familiar or unfamiliar voices evoked the higher signal depended on the task. Unfamiliar voices caused a higher signal during the voice condition when two voice samples had to be compared acoustically. In contrast, the familiar voices caused a higher signal during the sentence condition when there was no need to analyse vocal features in detail. Obviously, the necessity to acoustically compare voice samples raises the signal of unfamiliar voices more than the signal of familiar voices. In contrast, explicit and automated identification processes result in an activation preference for familiar voices. Altogether, all cited studies noticed that the temporal lobes discriminate between familiar and unfamiliar voices. Additionally, most of them could also show that familiar voices elicit a higher signal than unfamiliar voices. Yet, this seems to be the case only if familiarity is actively or automatically perceived by the listener and not overwritten by task demands to acoustically compare voice samples.

**Figure 5:**
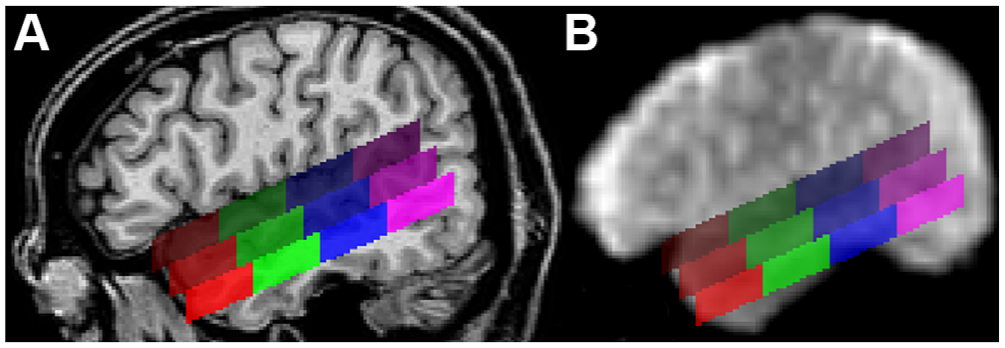
Example for the position of the regions of interest. (A) The sagittal view of a single subject’s brain shows the position of the ROIs along the superior temporal sulcus at 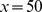 (right hemisphere). The upper row of ROIs covers the superior part of the STG, the middle row the inferior part of the STG, and the lower row the superior part of the MTG. Red ROIs are located in the anterior temporal lobe, green ROIs in the mid-anterior part, blue ROIs in the mid-posterior part, and purple ROIs in the posterior temporal lobe. The mean Talairach coordinates are given in Table 5. (B) Sagittal view (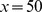) of the first functional EPI volume showing those brain regions that produced an MR signal.

**Table 5:** Position of all regions of interest in Talairach space.

	a		Ma		mp		p	
	LH	RH	LH	RH	LH	RH	LH	RH
**sSTG**	20; −12	20; −13	−01; −01	−01; −01	−20; 11	−20; 11	−40; 22	−40; 24
**iSTG**	14; −19	14; −20	−06; −08	−06; −08	−26; 03	−26; 05	−46; 15	−46; 17
**sMTG**	10; −26	10; −27	−10; −15	−10; −15	−30; −04	−30; −02	−50; 08	−50; 10

Given are the centres of the ROIs across all subjects as mean Talairach coordinates (y; z). The mean x-coordinate was 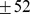. Abbreviations: a, anterior; iSTG, inferior part of the STG; LH, left hemisphere; ma, mid-anterior; mp, midposterior; p, posterior; RH, right hemisphere; sMTG, superior part of the MTG; sSTG, superior part of the STG.

Because of the differences in the BOLD response to familiar and unfamiliar voices in the temporal cortices, it seems reasonable to assume that these areas are specifically involved in voice processing. Particularly those portions of the temporal lobes that are located along the STS were repeatedly found to be activated when participants were presented with sounds that were produced by the human vocal folds [2], [5], [7], [8], [15]. The current study repeated these findings by showing that the processing of highly familiar voices evoked neural activity around the STS when these stimuli were compared to a baseline period without stimulation. As the functional data were not spatially smoothed, several activation clusters could be identified all along the STS in each single subject. However, the results of those studies that compared voices to rest periods, visual stimuli, or non-linguistic sounds are also compatible with the assumption that the identified areas have a part in linguistic (e.g., phonetic) processing regardless of any vocal features [9], [10], [12], [34], [35]. Likewise, the activation could merely reflect processes of acoustic analysis as noise and tones evoked neural activity in these parts of the temporal lobe as well [9], [11], [12]. Yet, although linguistic and acoustic processing cannot be ruled out as additive components, a specific role of the areas along the STS in voice recognition is most likely when the observation is taken into account that the activation signal was higher to familiar than to unfamiliar voices [23, 25, present study]. Further hints at the specific contribution of the temporal lobes to voice processing arose from studies that directly compared voice processing to the processing of linguistic, emotional, or directional information also present in vocal stimuli. These studies consistently found that areas surrounding the STS responded with a higher signal to the very same stimuli when the subjects focussed their attention on the vocal features and not on the other aspects [1], [6], [18]–[22], [36].

### Familiar and Unfamiliar Voices are Best Distinguished by Areas Around the STS and in the ATL

Even if all analysed parts of the temporal lobes produced a higher BOLD signal to familiar than to unfamiliar voices in the current study, it was areas along the STS and in the anterior temporal cortex that distinguished most clearly between these voice categories. Cortical regions around the STS were frequently reported to be activated by fMRI designs probing voice perception, independent of the task in hand. Belin et al. [7], for example, noticed activation clusters all along the STS when voices were compared to divers environmental sounds, Fecteau et al. [8] when voices were compared to animal vocalisations, Blank et al. [18] when the analysis of the vocal features was contrasted with an analysis of the linguistic content of spoken phrases, Andics et al. [37] and Latinus et al. [24] when different voice samples were presented in comparison to the repetition of one voice, and von Kriegstein et al. [23] when familiar voices were contrasted with unfamiliar voices. Thus, areas surrounding the STS seem to be most relevant to voice processing. By contrast, the importance of the anterior temporal lobe for voice recognition was mainly detected by studies that examined top-down oriented voice perception by comparing the processing of vocal features with the processing of linguistic or emotional information also contained in the spoken material [1], [19], [21], [36].

Yet, there is still much uncertainty about the precise function of these areas in voice recognition. Belin et al. [38] adapted the face processing model by Bruce & Young [39] to explain voice processing and differentiated between at least two major stages – the structural analysis of voices based on their acoustic features and their recognition as being spoken by a familiar person. Similarly, it was observed that brain-damaged subjects with voice recognition disorders fall in two main groups – one group with problems in voice discrimination tasks (apperceptive phonagnosia) and another group with deficits in the recognition of familiar voices and the identification of speakers (associative phonagnosia) [40]–[42]. The current experiments did not provide evidence for a functional dissociation between the anterior temporal lobes and more posterior parts of the STS as the BOLD time course was quite similar in both areas. Instead, the results argue for a contribution of both the anterior and the posterior areas around the STS in speaker identification because of the observed influence of voice familiarity on the BOLD signal. Others, however, argued for a more acoustically based function of the ‘voice areas’ [24] or for distinct mechanisms in posterior and anterior areas [22], [43]. The function of the anterior and more posterior portions of the STS in voice processing will now be discussed separately.

#### Posterior and central STS

More posterior areas surrounding the STS were mainly assumed to be engaged in acoustic-based voice processing. Von Kriegstein & Giraud [22] came to that conclusion because areas around the posterior STS produced a stronger BOLD signal to unfamiliar than to familiar voices, because the region was active even if the subjects focussed on the linguistic content of the spoken stimuli, and because that area was additionally found to be activated in response to meaningless sounds. Further studies argued for acoustic voice processing in central and posterior portions of the STS by showing that experimental conditions involving voice repetition resulted in a lower activation than the presentation of different voices or several variants of the same voice and as there were no signal differences between the latter two conditions [24], [37], [43]. A final piece of evidence is the observation that neural activity in response to voice perception was located in these areas mainly if voices were contrasted with acoustically rather dissimilar sounds as tones, scrambled voices, and meaningless or environmental sounds [2], [4], [6]–[8]. In contrast, activation clusters were less often found in these parts of the brain when the same voices were presented under different task demands (but see [18], [20]). However, as the current study revealed a stronger signal to familiar than to unfamiliar voices exactly in these posterior regions of the STS that were assumed to perform acoustic analyses, speaker identification processes seem to have some additional effect on the neural processing in these areas. Possibly, voice familiarity affects the neural response in these regions in a retroactive, top-down oriented manner.

#### Anterior temporal lobe

Regarding the anterior temporal lobes, there is consensus that these parts are involved in later steps of the voice recognition process. Firstly, in contrast to the central and posterior STS, the ATL was not identified as being voice-specific by designs that compared voices and other sounds, but by experiments in which the same spoken utterances were presented and had to be analysed either in respect to their vocal or their linguistic and emotional features [1], [19], [21], [36]. This finding suggests that the ATL performs a very detailed analysis of vocal features, a prerequisite for speaker identification. Secondly, associative phonagnosia is often observed in patients with lesions or atrophy in the ATL [31]–[33], [44]–[46]. Two such studies explicitly confirmed that their participants had no deficits in the acoustic processing of voices, e.g., by demonstrating intact voice discrimination abilities [40], [47]. Thirdly, fMRI studies on familiar and unfamiliar voice processing also suggest that speaker identification is sustained by cortical structures in the ATL. Several designs probing speaker identification elicited activation in the anterior temporal cortex, for instance, when subjects performed familiarity decisions [19], when voices had to be identified as being from one particular speaker [22], or when familiar voices were compared with unfamiliar voices [23, 25, present study].

Two recent fMRI studies directly aimed at disentangling apperceptive and associative stages in voice processing [24], [37]. Both used a training paradigm on a continuum of synthetic voices, which were generated by morphing the voices of unfamiliar speakers. In this way, experimental conditions were created that manipulated either the acoustic distance between samples or the identity of the presented voices. This allowed comparing the neural response to mere repetitions of a target voice, to acoustic variations of a target voice, and to the presentation of different voices. Latinus et al. [24] hypothesised that only those areas could be assumed to be relevant to speaker identification that show a larger signal to different voices than to variants of one voice. As the STS did not show that pattern, the authors concluded that these areas are involved in an acoustic-based representation of voices. Yet, although the subjects had to pass a voice learning task between the two MR sessions, during scanning a same-different discrimination task was requested that required to ignore the newly learned voice identity boundaries and to achieve the task by a purely acoustic analysis. Accordingly, the testing yielded identical performance scores before and after learning. If learning had had some effect on the task, the discrimination of voice variants would have dropped because they would have been perceived as identical. Therefore, the study is not very informative regarding the differentiation between apperceptive and associative stages in voice processing. In contrast, Andics et al. [37] explicitly used a speaker identification task and found that different voices caused a stronger activation than voice variants in the anterior temporal lobes. Thus, it seems that the ATL is involved in processes that allow recognising speakers by their voices.

If the anterior temporal lobes are important sites for the recognition of speakers, they could operate in a modality-specific (auditory) or multimodal manner such that faces and names of familiar people are also processed by these neural structures. Actually, many studies showed that the anterior temporal cortex is also engaged in the processing of familiar faces. On the one hand, face processing is often disrupted in patients with lesions of the ATL [48]–[56] and on the other hand, there are several reports on healthy subjects activating the ATL more when famous faces were recognized than when unfamiliar faces were presented [57]–[63]. In support of a multimodal function of the ATL in person recognition, two parallel studies observed stronger activation in the anterior temporal cortex when a familiarity decision task was compared with a control task on unfamiliar stimuli. This activation pattern was found regardless of modality, i.e., when faces were used as stimuli or when voices were used [19], [64]. Moreover, the ATL also responded with strong neural activity when the names of famous or personally known people had to be processed [58], [65]–[67].

Still other studies suggested that the anterior temporal lobes are not only relevant for person recognition but for the processing of all kinds of unique entities. Objects are processed as unique entities when these are recognised as particular individuals and not as categories of objects. Accordingly, the processing of non-human individuals (mainly places and buildings) was also found to elicit specific activation in the ATL [59], [61], [68] and to be disrupted by lesions of these brain regions [31], [42], [44], [46], [69]–[72]. Therefore, the necessity to process people and objects at an individual level could be the relevant factor for the activation in anterior temporal areas. This hypothesis was tested by us in two further experiments, which will be described in a future manuscript.

#### Voices, the temporal lobes, and social cognition

It has been argued that areas along the STS and in the ATLs are heavily involved in voice processing. Yet, this does not mean that these areas are specialised for voices. Instead, the observed neural activity could have been evoked by a specific way of processing (e.g. unique-level processing) or the processing of some essential features. These are physical stimulus features like acoustic parameters, but also semantic features. In the case of voices, a substantial portion of the semantic features consists of social features, i.e. those that describe psychological characteristics and that reveal socially relevant content. Thus, the reason that voices evoke temporal lobe activity might be that voices initiate the recognition of familiar persons, which gives rise to the retrieval of social features. Moreover, neural activity is expected to be higher for familiar than for unfamiliar voices because familiar persons should be associated with more social features.

Access to social knowledge, which allows us to interact with other persons, to recognize their thoughts and feelings, and to predict their reactions, is assumed to rely on areas along the STS and in the ATLs [73], [74]. The posterior STS was repeatedly observed to be activated when subjects trace the eye or reaching movements of other people [73], [75], which is an important capacity in order to infer somebody’s thoughts and feelings. The ATLs, in contrast, were proposed to be a store for the social knowledge itself, which includes knowledge about the world, about the person somebody interacts with, about the course of social situations, about how people respond to specific situations, and about how feelings and attitudes influence the behaviour of people. It was observed that fMRI tasks testing the subjects’ *theory of mind* evoked activation in the ATLs [76]–[78] and also the contrast between social and non-social concepts (*honourable* vs. *nutritious*) [78], [79]. Moreover, ATL lesions were found to cause deficits in social behaviour [80]–[82]. Often, these patients present with person recognition deficits.

Hence, these results suggest that the STS and the ATLs are involved in voice recognition because human beings are defined by social features, which are processed in these temporal regions. Further evidence (see [83]–[85]) for that hypothesis emerged from studies in autistic subjects whose major deficit concerns social interaction. Firstly, anatomical studies revealed structural abnormalities in areas along the STS. Secondly, hypoperfusion in the temporal lobes of autistic children was found. Thirdly, it was observed that the patients activated the STS to a different degree than healthy control subjects when social cognition was probed. And fourthly, voice processing did not trigger activation in the classical voice areas around the STS.

### Hierarchical Processing of Voices Along the Antero-posterior Axis of the Superior Temporal Lobe

As mentioned before, the BOLD signal in the present experiments was higher to familiar than to unfamiliar voices in all analysed parts of the temporal lobes and the difference was largest in areas around the STS and in the ATL. In ROIs that covered the primary and secondary auditory cortices, the difference was smallest. In between, there was a gradual change from superior to inferior ROIs and from central-posterior to anterior and to even more posterior ROIs such that highly familiar and unfamiliar voices were increasingly better distinguished by areas increasingly more distant from the early auditory cortices. The most prominent gradient observed ran from the transverse temporal gyri towards the temporal pole. This pattern is in good agreement to previous reports that described a hierarchical organisation in the temporal lobes such that the processing of human and animal vocalisations is distributed along an antero-posterior axis [22], [43], [86]. According to von Kriegstein & Giraud [22], ‘segregated cortical regions along the STS are involved in distinct aspects of voice processing’ and Warren et al. [43] assumed that ‘abstraction of voice identity features occurs in posterior superior temporal sulcus, and further analysis of voice information occurs in anterior superior temporal sulcus and higher order cortices in the middle and anterior temporal lobe’.

As is reported in the previous section, there are clues on a functional dissociation between apperceptive and associative processing stages. Yet, because of the gradual signal changes we have observed, we would not assume that these functions are supported by completely distinct temporal areas. Instead, we believe that a model as the convergence zone theory by Damasio [87] is well suited to explain our results. The model assumes that characterising features of objects (and also of people) are distributively represented in early sensory, motor and affective brain structures. The integration of these features to holistic concepts is achieved by multiple stations, called convergence zones, which are organised hierarchically. Initially, tiny fragments of objects are processed in early sensorimotor areas. Then, these fragments become integrated in local modality-specific zones whose information is assembled by still higher zones in modality-independent association areas. An important integration pathway is assumed to run from posterior brain regions towards the temporal pole (see also [88], [89]). Regarding the present results, the model could be interpreted such that early and local convergence zones in auditory cortices analyse acoustic features relevant for voices of all familiarity grades. Towards the anterior temporal cortex, increasingly more acoustic and possibly also non-acoustic features are assembled. Since concepts of familiar people contain more biographical features than representations of unfamiliar people, the signal difference between well and less known voices should increase together with the number of convergence processes, which is higher the further anterior a convergence zone is located. This is exactly what could be observed. Anterior temporal areas differentiated more clearly between familiar and unfamiliar voices than areas in early auditory cortices.

## Materials and Methods

### Participants

A total of 31 young adults who were native German speakers participated in the present study on a voluntary basis. None of the participants reported any history or evidence of neurological, psychiatric, or audiological symptoms. All gave written informed consent according to local institutional guidelines and were paid a small hourly stipend. The study received prior approval by the ethics committee of the Otto von Guericke University Magdeburg, Germany.

16 subjects participated in Experiment 1. In three of them, the response behaviour did not match the instructions leaving 13 data sets for analysis. The mean age of these 13 right-handed participants (8 women) was 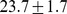 years (mean ± standard deviation). 24 subjects volunteered for Experiment 2. Here, two data sets had to be discarded from the analysis because of strong head motion. Five of the resulting 22 subjects had also participated in the first experiment with a time span of at least six months between the two sessions. The mean age of the 22 right-handed participants (14 women) was 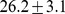 years.

### Experimental Designs

#### Stimuli

The subjects were presented binaurally with auditory stimuli (44 100 Hz, 16 bit, mono) which were utterances spoken spontaneously by famous or unknown German people. The utterances had a duration of 2 s and consisted of several consecutive words forming short phrases. The excerpts were chosen such that the content gave no hint as to the identity of a speaker. The utterances were extracted from video clips published on the websites of public German broadcasting corporations. They were recorded and processed using the software Cool Edit 2000 (Syntrillium Software Corporation, Phoenix, USA).

75 utterances were used in Experiment 1 with 50 utterances being spoken by famous people (16 women) and 25 by unknown people (8 women). In Experiment 2, 80 utterances were used with 70 utterances being spoken by famous individuals (27 women) and 10 by unknown individuals (4 women). By the higher number of famous than unfamiliar voices, we hoped to enhance the number of identified people because it is known from behavioural studies that voice recognition is often very low [26], [29], [30], [90]. Across both experiments, there was a common set of 46 famous speakers. Within each experiment, each speaker was presented only once.

#### Timing

The stimuli were presented using a slow event-related fMRI design with a long rest period after each utterance. The rest period had a duration of 12 s in Experiment 1 and of 16 s in Experiment 2.

#### Tasks

The subjects’ task was to identify the speakers of the utterances and to indicate their familiarity with a presented voice after each stimulus presentation. In Experiment 1, the subjects had to accomplish the task by pressing one of two buttons. At first, they were asked to indicate whether or not they were familiar with the voice (familiar  =  index finger, unfamiliar =  middle finger). Then, in case of familiarity, they had to specify whether they were able to name the speaker (index finger) or not (middle finger). In case of unfamiliarity, they were requested to classify the voice according to its gender (male =  index finger, female =  middle finger). Using this procedure, each stimulus was responded to twice.

In Experiment 2, the responses had mainly to be given by overt speaking. The participants were asked to indicate their familiarity with a voice by one of four responses. First, they had to declare unfamiliarity when the voice was unfamiliar to them. Second, they had to declare familiarity when a voice caused some feeling of familiarity but no further detail about the speaker could be given. Third, the speaker had to be named when he or she could be identified. And fourth, in case of name retrieval failures, subjects were requested to describe the identified speaker with short comments on their biography, appearance or other identifying features. To keep head motion to a minimum, the time for overt speaking was restricted by a tone to 4 seconds. After the tone, the participants were only allowed to respond by pressing a button. With the index finger they could upgrade stimuli previously classified as being unfamiliar or familiar or those that were semantically described and indicate thereby that a name was suddenly available. With the middle finger, stimuli previously classified as being unfamiliar or familiar could be upgraded indicating that biographical information was available right now. This procedure was chosen because it was assumed that response inhibition would evoke more unwanted neural activity than giving the information by pressing a button.

The aim of both experiments was to distinguish the neural responses to as many familiarity levels as possible. For that reason, the subjects were asked to specify very precisely the amount of information they were able to retrieve from each person they had listened to. The experiments used different procedures to gather the information. Responses had to be given by means of a keypad in Experiment 1 and by overt speaking in Experiment 2. Both procedures have their advantages and disadvantages. Responding with a keypad is preferred in fMRI studies to reduce head motion, but it was expected to induce unwanted meta-reflections about how to respond even with a few choices. Overt speaking, in contrast, is a more natural way of responding and thereby more effective in separating different familiarity levels. Thus, Experiment 1 distinguished between unfamiliar, familiar, and named voices only, whereas Experiment 2 allowed to further separate familiar, non-named voices into familiar-only and associated voices as well as named voices into slowly and quickly named voices. The latter was not possible in Experiment 1 because the subjects were first required to indicate familiarity and only then to signal successful name retrieval.

### Imaging Methods

#### Data acquisition

Magnetic resonance imaging was conducted at a 3 T scanner (Siemens, Erlangen, Germany) using a head array receive coil with eight channels. Stimulus presentation was timed by the software Presentation 9.20 (Neurobehavioral Systems, Inc., Albany, USA), which was also employed for the recording of the responses given by the subjects via response buttons. For stimulus presentation, MR-compatible headphones with integrated dual-channel microphones were used (MR confon, Magdeburg, Germany) adjusted to a comfortable listening level. The spoken responses were recorded by the software Cool Edit 2000 (Syntrillium Software Corporation, Phoenix, USA) running on a separate notebook. To protect the participants against the scanner noises, they wore ear plugs. The participants kept their eyes closed during all scans.

In each subject, three scan sequences were performed. At first, high-resolution T_1_-weighted images with 1 mm isotropic resolution were acquired using an MPRAGE sequence (192 gapless axially oriented slices, field of view = 256×256 mm^2^, TR = 2500 ms, TE = 4.77 ms, TI = 1100 ms). The scan covered the whole brain and served to reconstruct the individual three-dimensional brain anatomy. Secondly, a T_1_-weighted, anatomical, two-dimensional data set was acquired with an IR-EPI sequence (TR = 20 000 ms, TE = 34 ms, TI = 1450 ms). Other parameters as orientation and geometry were equal to the functional scans which were done in a last step. The functional images were taken using a T_2_-weighted GE-EPI sequence (32 axially oriented slices, voxel size = 3×3×3 mm^3^, interslice gap = 0.3 mm, field of view = 192×192 mm^2^, matrix = 256×256 voxels, TR = 2000 ms, TE = 30 ms, TI = 62 ms, flip angle = 80°). Both 2D image sets were oriented roughly parallel to the sylvian fissure with only minor differences between the subjects to ensure maximal coverage of the entire cerebrum, excluding only the most superior frontoparietal regions and parts of the occipital lobes.

Experiment 1 was acquired in 17 min 44 s and resulted in 532 volumes, Experiment 2 was acquired in 24 min 24 s and resulted in 734 volumes. Each examination took less than one hour.

#### Data preprocessing

All processing steps and the analysis of the MRI data were done using the BrainVoyager QX software, version 1.8.6 (Brain Innovation, Maastricht, The Netherlands). The anatomical 3D data were transformed into AC-PC and Talairach standard space [91]. After having imported the functional data, a standard sequence of preprocessing steps was applied, including slice scan time correction, head motion correction, linear trend removal, and temporal highpass filtering with two cycles per scan. No spatial smoothing was done. Finally, the three- and two-dimensional data sets were registered to display activations in 3D space.

Additionally, the functional data were inspected thoroughly for severe grey level fluctuations resulting from head motion. For that purpose, the automated head motion correction procedure, which resulted in estimated translation and rotation parameters for each spatial direction, was analysed. Data sets with parameters that exceeded 3 mm or 3° were excluded. Then, the data were checked for smaller jerky movements as these can also lead to signal artefacts. A jerky move was defined as a translation or rotation of the head from one volume to the next in the magnitude of 0.5 mm or 0.5° in one spatial direction or of 1.0 as the sum of all directions. A data set was discarded from the analysis when these sudden movements occurred at least ten times. Otherwise, the respective volumes were eliminated to correct for outliers.

### Analysis

#### Conditions

The experimental conditions were defined with reference to the different degrees of voice familiarity. Since voice familiarity is subject to personal knowledge, voices were not assigned *a priori* to a particular condition but individually on the basis of a subject’s response pattern. For that reason, the chronological order and the frequency of the conditions differed between the participants. The number of the conditions, however, was constant across all subjects within one experiment.

Experiment 1 resulted in three conditions: unfamiliar (U), familiar (F), and named (N) voices. Unfamiliar voices were those that were classified as being unfamiliar. Familiar voices were those that were rated as being familiar, but could not be named. Moreover, voices spoken by unknown people that were categorized as being named (which actually happened very seldom) were grouped together with the familiar stimuli. Named voices were those that belonged to famous people and for which subjects indicated successful name retrieval.

Experiment 2 generated four conditions: unfamiliar (U), familiar (F), associated (A), and named (N) voices. Unfamiliar voices were again those that were classified as being unfamiliar. Familiar voices were those that were categorised as being familiar but could not be named or semantically described. Semantically associated voices included those for which a semantic description was given (independent of detail and correctness), those that were labelled with an incorrect name, and those that were initially classified as unfamiliar or familiar or were described and later responded to with a button press to indicate subsequent semantic access or name retrieval. Named voices were those that were overtly denoted with the correct name. Due to the immediate spoken responses, the named voices could additionally be subdivided into voices that were named quickly (N_Q_) and those that were named slowly (N_S_). The subdivision was done for each participant separately and was based on the individual response times. The response time was the period from the beginning of a stimulus to the onset of the correct spoken response. The statistical median of all naming times of a subject was used to separate quick and slow responses.

#### Activation maps

In each participant, a parametric activation map was generated by applying a general linear model to each voxel. The model was convolved with the canonical two gamma hemodynamic response function using the following parameters: response peak at 5 s after stimulus begin, undershoot peak at 15 s after stimulus begin; the relation between minimum and maximum was 6. Predictor variables of the estimated time course were the three or four conditions of an experiment but also the head motion parameters that had been identified with the head motion detection procedure. These were included to weight and reduce the influence of smaller head motion on the signal change.

The condition *named voices* was defined as main predictor. By contrasting this condition with the rest period, the activation map showed those voxels that resulted in significant activation during speaker recognition. Of these voxels those were analysed that were significant at a level of 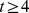 (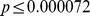) and that formed a cluster of at least four adjacent functional voxels (each having a size of 3×3×3 mm^3^). To reduce signal artefacts from brain areas with low signal intensity, only those voxels were considered whose functional EPI signal had a grey level of at least 75. The maps were not spatially smoothed.

#### ROI analysis

Two regions of interest (ROIs) were created to measure the extent of activation in the left and right temporal lobe. These ROIs were created individually in each participant using their structural MRI data. The position of the ROIs was aligned to the slope of the superior temporal sulcus (STS) along the y-axis of the brain from anterior to posterior coordinates. As the slope of the STS alters from lateral to more medial slices, the slope of the ROIs was adjusted accordingly. Nine consecutive sagittal ROI slices each had an identical slope, with four voxels left and right of 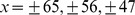, and 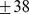. This was done separately for each hemisphere. In all, the ROIs extended from lateral 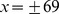 to medial 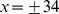.

The ROIs were bilaterally subdivided into 12 subregions with three rows and four columns (Figure 5). Each row had a height of ten voxels (i.e., 10 mm). The upper row covered the superior part of the STG (*sSTG*), the middle row the inferior part of the STG (*iSTG*), and the lower row the superior part of the MTG (*sMTG*). Each row was further subdivided into four ROIs; an anterior ROI (*a*), a central-anterior ROI (*ca*), a central-posterior ROI (*cp*), and a posterior ROI (*p*). The upper row ran from anterior 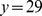 to posterior 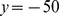 (*a* with 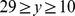; *ca* with 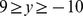; *cp* with 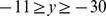; *p* with 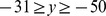). Compared to the upper row, the middle row was moved backwards by five voxels, the lower row by 10 voxels. Each of the 24 ROIs was composed of 7200 voxels (voxel size = 1 mm^3^). Their centres are given in Table 5.

#### Extracted values

From each of the 24 ROIs, the mean BOLD signal change during each condition and the number of the significantly activated voxels were extracted for further statistical analysis. The baseline was calculated from three time points. These were the two time points just before stimulus presentation and one time point during presentation. Each time point had a duration of 2 s, which was the time to scan the whole brain once ( =  one brain volume). Since the BOLD signal typically does not start to rise before two seconds after stimulus begin and as stimulus presentation took two seconds, the time point during stimulus presentation could be integrated into the baseline period. From the resulting signal time course, one BOLD value for each condition was extracted. This value was the mean of the BOLD intensities during scanning the third to the sixth brain volume after stimulus onset.

The BOLD values were further used to compute the BOLD ratio of named (N) to unfamiliar (U) and familiar (F) voices (Equation 1). These were the voice categories that triggered the largest signal differences with named voices eliciting the highest intensities and unfamiliar as well as familiar voices eliciting the lowest intensities. If this is true and if all conditions evoke positive activation, the resultant ratio ranges between 0 and 100 with higher values reflecting larger signal differences.
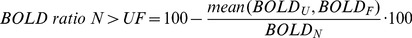
(1)


#### Statistics

The statistical analysis was performed using the software package IBM SPSS Statistics (IBM Corporation, New York, USA). At first, the data were checked for normal distribution with the Shapiro-Wilk test. As normal distribution could not be confirmed in various series, non-parametric tests were utilized for further analyses. Because of the repeated-measures design (within subjects), this was the Friedman test as omnibus test and the Wilcoxon signed-rank test as post hoc test. The *p* values from the pairwise comparisons of the Wilcoxon test were Bonferroni adjusted to counteract an inflation of the familywise error rate (e.g. 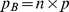, with *n* being the number of comparisons). The results were always given for two-tailed testing. Raw scores were presented as mean ± standard error of the mean.
